# The Effect of Hyperuricemia on Cognitive Impairment: A Cohort Study and Systematic Review

**DOI:** 10.3390/nu18111813

**Published:** 2026-06-04

**Authors:** Ruei-Ting Su, Jerry Cheng-Yen Lai, Pei-Yu Wu

**Affiliations:** 1 Department of Nutrition, Taitung MacKay Memorial Hospital, Taitung City 950, Taiwan; ag082@mmh.org.tw; 2 Master Program in Biomedicine, College of Science and Engineering, National Taitung University, Taitung City 950, Taiwan; chengyen@gm.nttu.edu.tw; 3 Department of Nutrition, China Medical University, No. 100, Section 1, Jingmao Road, Beitun District, Taichung City 406040, Taiwan

**Keywords:** hyperuricemia, cognitive impairment, cohort study, systematic review

## Abstract

Background/Objectives: Hyperuricemia may influence cognitive function, but current evidence remains inconsistent. This study examined the association between hyperuricemia/gout and cognitive impairment through a prospective cohort study and a systematic review of cohort studies. Methods: Data from 1959 Taiwan Biobank participants aged ≥60 years without mild cognitive impairment (MCI) or dementia at baseline were analyzed over a mean follow-up duration of 4.47 years (2012–2021). Cognitive function was assessed using the Mini-Mental State Examination (MMSE). Participants were classified by changes in serum uric acid status from baseline to follow-up, and Cox proportional hazards models were used to estimate hazard ratios (HRs) and 95% confidence intervals (CIs). For the systematic review, cohort studies published up to March 2026 were identified from eight databases. Results: During follow-up, 1013 participants developed incident MCI and 132 developed dementia. Compared with participants who maintained normal serum uric acid levels, those who developed hyperuricemia during follow-up had a significantly lower MCI risk (HR = 0.72, 95% CI = 0.57–0.90), as did those with persistent hyperuricemia (HR = 0.78, 95% CI = 0.64–0.95). No significant association was observed for dementia. The systematic review of five prospective cohort studies comprising 2,261,704 participants showed inconsistent findings with considerable heterogeneity (*I*^2^ = 96.7%). Conclusions: Rising serum uric acid levels were associated with lower MCI risk, but not dementia. These findings should not be interpreted as support for intentionally increasing serum uric acid levels or withholding urate-lowering therapy. Further long-term studies are needed.

## 1. Introduction

Dementia is associated with long-term cognitive decline and is considered one of the greatest health and social care challenges of the 21st century. In 2019, the global prevalence of dementia was 57.4 million people, and this is projected to increase to 158.2 million people by 2050 [1]. According to the World Health Organization, approximately 55 million people worldwide are living with dementia, with nearly 10 million new cases occurring annually, primarily due to population aging [2]. Global dementia-related expenditure was estimated at US$948 billion in 2016, increasing by 15.9% annually from 2000 to 2016 [3]. In 2019, the total global cost of dementia reached US$1.3 trillion [4].

Hyperuricemia is an independent risk factor for gout, diabetes, cardiovascular disease, and chronic kidney disease [5]. Persistent hyperuricemia can lead to urate deposition, resulting in tissue and organ damage. Gout is the most common inflammatory arthritis in adults, and hyperuricemia is included in its clinical diagnostic criteria [6]. Uric acid is the final product of purine nucleotides and promotes both oxidation and anti-oxidation function. Serum uric acid levels may be influenced by dietary factors, including purine-rich foods, fructose, sugar-sweetened beverages, and alcohol, which can increase uric acid production or reduce renal excretion [7]. Uric acid is involved in the oxidative stress and inflammation process and may affect not only the joints but cognitive function [8].

To date, three systematic reviews and meta-analyses have examined the association between hyperuricemia and cognitive impairment. A meta-analysis of four cohort studies reported no association between hyperuricemia and dementia [9]. However, the definition of hyperuricemia used in the included studies, namely 360 µmol/L for men and 300 µmol/L for women, was lower than the recommended clinical guideline thresholds of 416.36 µmol/L (7 mg/dL) for men and 356.88 µmol/L (6 mg/dL) for women. In addition, the mean age of participants in the included studies was older than 60 years. More recently, a cohort study [10] and meta-analysis [11,12] suggested that gout or hyperuricemia may be associated with a reduced risk of dementia. Wang and colleagues conducted a meta-analysis of seven cohort studies, three of which included participants with a mean age younger than 65 years [11]. This meta-analysis focused on Alzheimer’s disease. All included studies either used diagnostic criteria for gout or defined hyperuricemia as serum uric acid levels greater than 360 µmol/L for men and 300 µmol/L for women. Rabbani and colleagues focused on the association between serum uric acid and different domains of cognitive function [12]. Variations in the definition of hyperuricemia and participant age ranges across studies may contribute to inconsistent findings. Therefore, an updated systematic review and meta-analysis including studies that define hyperuricemia according to clinical guideline recommendations, with subgroup analyses by older age, may be necessary to clarify the association between hyperuricemia and cognitive function.

Moreover, most previous studies have investigated the risk associated with hyperuricemia, whereas few have examined whether the restoration of hyperuricemia to the normal range alters the risk of cognitive impairment. To the best of our knowledge, only two studies have documented the association between hyperuricemia and cognitive impairment risk [10], and no study has specifically focused on older adults. Aging is a major risk factor for many chronic diseases and disorders, including cognitive impairment and dementia. Older adults have a higher prevalence of cognitive impairment and dementia than younger populations. Therefore, the present study aimed to examine the effects of transitions from hyperuricemia to normal uric acid levels, and vice versa, on the risks of cognitive impairment and dementia in older adults.

## 2. Materials and Methods

### 2.1. Cohort Study

The Taiwan Biobank is a prospective cohort study designed to advance epidemiological and biomedical research. Detailed information on the Taiwan Biobank has been reported previously [13]. Since 2012, the database has recruited participants aged 20–70 years from the general Taiwanese population, with follow-up data collected from 2019 to 2021 [14]. All participants had no history of cancer at enrollment, provided informed consent, and underwent physiological measurements as well as blood and urine testing at baseline and follow-up. Trained researchers conducted one-on-one interviews and measurements using standardized questionnaires and equipment. Blood pressure and biochemical measurements, including serum uric acid, HbA1c, HDL-cholesterol, triglycerides, and fasting glucose, were performed according to the standardized protocols of the Taiwan Biobank, as described previously [13,14]. The validity of the Taiwan Biobank self-reported questionnaire has been evaluated using Taiwan’s National Health Insurance database, and several self-reported diseases, including dementia, depression, and coronary artery disease, were found to be highly correlated with claims records [15].

This study recruited participants with complete data on basic demographics, serum uric acid levels, and cognitive function assessments using the Mini-Mental State Examination (MMSE) at baseline and follow-up. Trained interviewers also collected medical history, including coronary artery disease, hypertension, diabetes, and smoking status. The study was reviewed and approved by the MacKay Memorial Hospital Institutional Review Board (original approval number: 19MMHIS405e, 5 December 2019; amended approval number: 21MMHIS351e, 22 December 2021). Because this study used a fully de-identified secondary database, the requirement for informed consent was waived in accordance with applicable regulations.

Participants were required to be 60 years or older and to have complete MMSE and serum uric acid data. Participants with a follow-up period of less than three years, incomplete follow-up data, baseline MMSE scores < 27, or a self-reported history of schizophrenia, dementia, alcoholism, or substance abuse were excluded.

Participants were classified into either the hyperuricemia group or the normal uric acid group at baseline and follow-up. Hyperuricemia was defined according to Chinese criteria as serum uric acid levels ≥ 6 mg/dL for women and ≥7.2 mg/dL for men [16], or according to US criteria as ≥6 mg/dL for women and ≥7 mg/dL for men [17]. The primary analysis used the Chinese criteria to define hyperuricemia. A sensitivity analysis was conducted to examine the effect of different hyperuricemia definitions on the results.

The MMSE consists of 11 items with a total score of 30 and assesses five domains: orientation, registration, attention and calculation, short-term recall, and language [18]. An MMSE score < 27 indicates mild cognitive impairment (MCI) [19]. Illiterate individuals with MMSE scores < 17, participants with elementary education (<6 years) with scores < 20, and participants with higher education with scores < 24 were considered to have dementia. The validity of these MMSE cutoff points has been confirmed in large-scale studies involving Chinese populations [20].

Statistical analyses were performed using SAS software version 9.4 (SAS Institute Inc., Cary, NC, USA). A *p* value < 0.05 was considered statistically significant. Student’s t test or chi-square test was used to compare age, MMSE scores, sex, education level, medical history, including coronary artery disease and diabetes, and smoking status between participants with hyperuricemia and those with normal uric acid levels at baseline. Cox proportional hazards models were used to determine whether baseline hyperuricemia increased the risk of dementia or MCI during follow-up. Participants were divided into four groups based on serum uric acid levels at baseline and follow-up: persistent hyperuricemia, hyperuricemia at baseline that normalized during follow-up, normal uric acid levels at baseline that progressed to hyperuricemia during follow-up, and persistently normal uric acid levels. Cox proportional hazards models were used to compare the risks of MCI and dementia among these four groups and to examine whether baseline hyperuricemia was associated with decreased MMSE scores. The Cox proportional hazards models were adjusted for risk factors for MCI or dementia [21,22] and for demographic variables that differed significantly between the hyperuricemia and normal uric acid groups at baseline. Three models were applied: Model 1 was unadjusted; Model 2 was adjusted for demographic risk factors, including age, sex, and education level; and Model 3 was additionally adjusted for lifestyle factors, including smoking status, living alone, overweight or obesity, and physical activity, as well as history of coronary artery disease, diabetes, and depression. Restricted cubic splines (RCS) were used to examine the dose–response association and linearity between serum uric acid levels and the risk of cognitive impairment in the Cox proportional hazards models. Five knots were placed at the 5th, 25th, 50th, 75th, and 95th percentiles, and the median serum uric acid level of 5.5 mg/dL was set as the reference. Mediation analysis was performed using Hayes’ PROCESS macro version 3.5 (Model 4) for SAS to evaluate the mediating effects of diastolic blood pressure, HbA1c, and serum triglycerides on the association between hyperuricemia and MCI risk [23]. These variables were selected based on biological plausibility and significant baseline differences between groups [24,25,26]. The temporal sequence was defined as hyperuricemia at baseline (exposure), mediators measured at baseline and follow-up, and incident MCI at follow-up (outcome). Age, sex, and education level were included as covariates to control for confounding in both the exposure–mediator and mediator–outcome relationships. A bootstrap distribution of 5000 samples was used to obtain bias-corrected 95% CIs for the indirect effects. Given the data-driven nature of mediator selection, all mediation results should be considered exploratory.

### 2.2. Systematic Review

The literature search was conducted up to March 2026 using the following eight electronic databases: PubMed, MEDLINE, Embase, Web of Science, Scopus, Cochrane Database of Systematic Reviews, Airiti Library, and the National Digital Library of Theses and Dissertations in Taiwan. The full search strategy for each database is provided in Supplementary Table S1. MeSH terms were used to expand the search vocabulary. References from review articles were also examined to identify additional relevant studies. To select eligible studies, two researchers (PYW and RTS) independently screened article titles, abstracts, and full texts. This study was conducted in accordance with the Preferred Reporting Items for Systematic Reviews and Meta-Analyses (PRISMA) statement [27].

The population, intervention, comparison, outcomes, and study design (PICOS) criteria were as follows: population, general population; intervention/exposure, diagnosis of gout according to standardized diagnostic criteria, such as International Classification of Diseases (ICD) versions 8–10, or hyperuricemia based on serum uric acid levels of ≥7.0 mg/dL (USA) or ≥7.2 mg/dL (China) for men and ≥6 mg/dL for women; comparison, normal serum uric acid levels; outcomes, diagnosis of any type of dementia according to standardized diagnostic criteria, such as ICD-8–10 or Diagnostic and Statistical Manual of Mental Disorders (DSM) III–V criteria, or MCI assessed using questionnaires such as the MMSE; and study design, cohort study.

**Figure 1:**
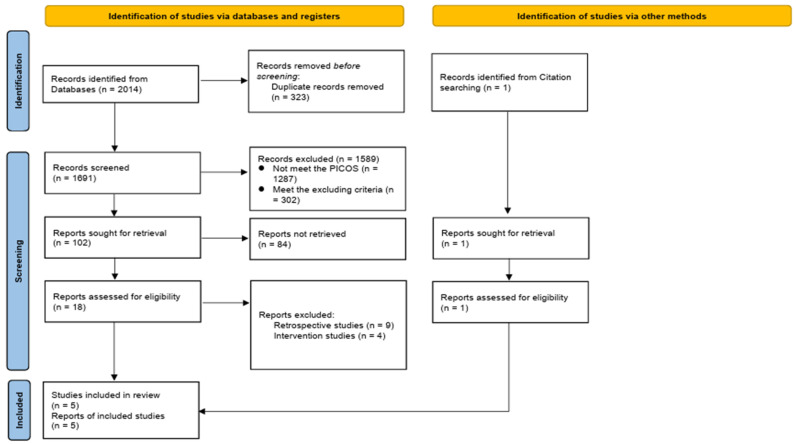
The flowchart of selected study in systematic review.

The screening criteria included impaired cognitive function indicated by abnormal cognitive assessment results. Impaired cognitive function was defined according to questionnaire scores, including the General Practitioner Assessment of Cognition, MMSE, and Geriatric Depression Scale. For example, a General Practitioner Assessment of Cognition score < 5 was considered indicative of cognitive impairment [28]. MMSE scores < 27 and <17–24 indicated MCI and dementia, respectively, depending on education level. Participants younger than 18 years at baseline were excluded. Articles not written in English or Chinese, non-peer-reviewed publications, conference abstracts, review articles, and errata were also excluded. If more than one article reported the same study, the article with the longest follow-up period was included in the systematic review. The study selection process is shown in Figure 1 according to the PRISMA 2020 flow diagram [27]. During the search, 5 cohort studies on hyperuricemia and dementia/cognitive impairment were identified. Studies in which serum uric acid thresholds were below the US or Chinese standards were excluded, leaving five studies for inclusion.

Two researchers (PYW and RTS) independently extracted data from each study using a standardized format. Extracted data included author, publication year, study location, sex, age, uric acid levels, diagnostic criteria, gout diagnosis, sample size, incidence of dementia or MCI, follow-up duration, and adjusted covariates. In cases of incomplete data, the corresponding author of the article was contacted by email for additional information.

Study quality was assessed using the Newcastle–Ottawa Scale [29]. Scores of 0–3 indicated low quality, 4–6 indicated moderate quality, and 7–9 indicated high quality. When studies reported models with different levels of covariate adjustment, the model with the most comprehensive adjustment was used. Two researchers (PYW and RTS) independently evaluated study quality. Any disagreement was resolved by a third researcher (JCYL).

All statistical assessments of heterogeneity were performed using Comprehensive Meta-Analysis (CMA) version 4.0 (Biostat Inc., Englewood, NJ, USA). Although substantial heterogeneity was observed (*I*^2^ = 96.7%, Q = 152.3, *p* < 0.001), the small number of included studies (*n* = 5) limited the feasibility of formal subgroup analyses, as each potential subgroup would contain only one or two studies, rendering the estimates statistically unreliable and vulnerable to sparse-data bias. Therefore, heterogeneity was explored through narrative synthesis.

## 3. Results

### 3.1. Cohort Study Results

**Figure 2:**
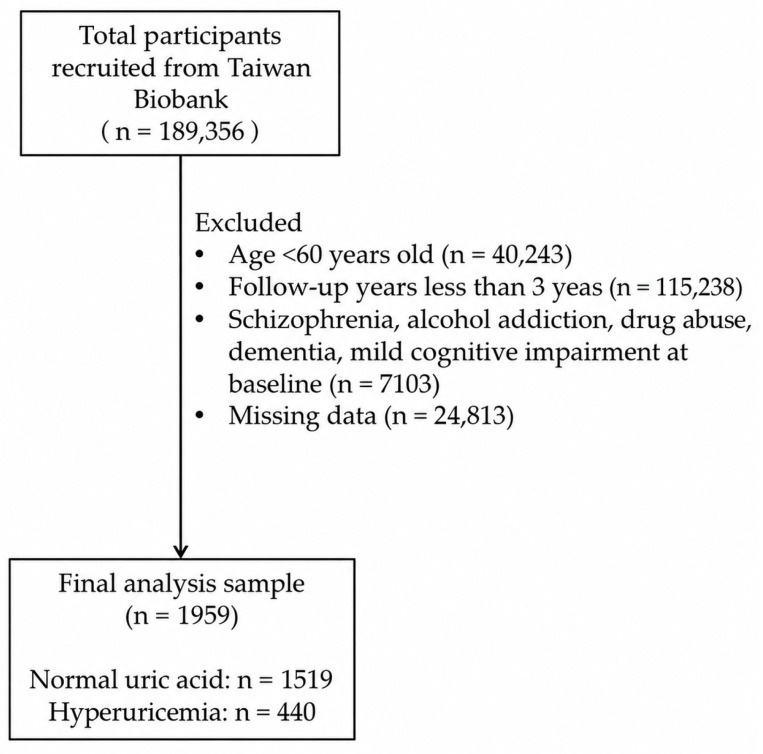
Flowchart of prospective study.

A total of 1959 participants were included in the analysis, and the flowchart of the analysis is shown in Figure 2. At baseline, the mean age was 64.43 ± 2.82 years, and 34.9% of participants were men. Participants were divided into two groups according to the presence or absence of hyperuricemia. Table 1 presents the baseline characteristics of the hyperuricemia and normal uric acid groups. The hyperuricemia group had significantly higher waist-to-hip ratio, body mass index, systolic blood pressure, diastolic blood pressure, triglycerides, and glycated hemoglobin levels. Conversely, their MMSE scores and serum high-density lipoprotein cholesterol level were significantly lower. In addition, the hyperuricemia group included higher proportions of female participants, participants with diabetes, and postmenopausal participants, but fewer participants who smoked and fewer participants with MCI. The distribution of education level also differed significantly between the hyperuricemia and normal uric acid groups. Other indicators did not differ significantly between the two groups.

The mean follow-up duration was 4.47 years. During follow-up, 1013 participants developed MCI, and 132 developed dementia. Participants with hyperuricemia had a significantly lower risk of MCI (*p* < 0.05). Participants were further divided into four groups according to changes in serum uric acid levels during follow-up: persistent hyperuricemia, hyperuricemia at baseline that normalized during follow-up, normal serum uric acid levels at baseline that progressed to hyperuricemia during follow-up, and persistently normal serum uric acid levels. Table 2 compares the incidence of MCI among these four groups during follow-up. Compared with participants who maintained normal serum uric acid levels throughout the study, the risk of MCI was significantly lower among those with normal serum uric acid levels at baseline who developed hyperuricemia during follow-up (HR = 0.72, 95% CI = 0.57–0.90, *p* = 0.005). A similar result was observed among participants with persistent hyperuricemia throughout the study period (HR = 0.78, 95% CI = 0.64–0.95, *p* = 0.014). However, after adjustment for age, sex, education level, smoking history, coronary artery disease, diabetes, depression, living alone, overweight or obesity, and physical activity, only the group with normal serum uric acid levels at baseline who developed hyperuricemia during follow-up continued to show a significantly lower risk of MCI (HR = 0.75, 95% CI = 0.59–0.94, *p* = 0.013).

Table 3 presents the sensitivity analysis results. Using the US definition of hyperuricemia did not materially affect the outcomes. In Model 3, only participants with normal serum uric acid levels at baseline who developed hyperuricemia during follow-up had a significantly lower risk of MCI than those who maintained normal serum uric acid levels (HR = 0.77, 95% CI = 0.62–0.96, *p* = 0.022).

**Figure 3:**
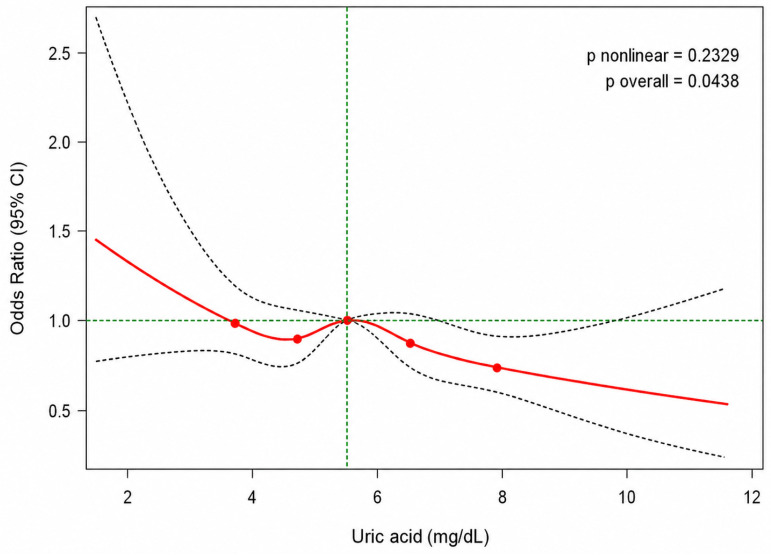
The association between serum uric acid levels and the risk of mild cognitive impairment. The odds ratios were estimated using serum uric acid of 5.5 mg/dL as the reference value. The model was adjusted for age, sex, education level, smoking status, living alone, overweight or obesity, physical activity, and history of coronary artery disease, diabetes, and depression. The red solid line indicates the estimated odds ratio, the black dashed lines indicate the 95% confidence interval, and the green dashed lines indicate the reference lines.

According to the multivariable-adjusted restricted cubic spline analysis (Figure 3), there was a significant dose–response association between serum uric acid levels and MCI (*p* for overall association = 0.0438). However, the results did not support a non-linear association between serum uric acid levels and MCI (*p* for non-linearity = 0.2329). The model was adjusted for age, sex, education level, smoking status, living alone, overweight or obesity, physical activity, and history of coronary artery disease, diabetes, and depression.

In the mediation analysis, diastolic blood pressure, HbA1c, and serum triglycerides did not significantly mediate the association between hyperuricemia and MCI. The effect estimates ± standard errors and *p* values were as follows: diastolic blood pressure, −0.0086 ± 0.0067 (*p* = 0.1962); HbA1c, 0.0002 ± 0.0027 (*p* = 0.9516); and serum triglycerides, −0.0165 ± 0.0096 (*p* = 0.0872).

### 3.2. Systematic Review Results

Table 4 summarizes the five included studies. These five prospective cohort studies were published between 2015 and 2023 and included a total of 2,261,704 participants. All participants were aged ≥40 years. Four studies included participants with a mean age older than 65 years at baseline [10,30,31,32], but only one study specifically focused on participants aged ≥65 years [32]. One study did not report mean age data [33]. All studies included both male and female participants. Three studies were conducted in Asia, specifically Singapore, Taiwan, and South Korea; one was conducted in the United States; and one was conducted in Europe, namely the United Kingdom. The mean follow-up duration was greater than 2.3 years.

In all studies, medical records were used to diagnose gout, except for one study in which gout diagnosis was self-reported [10]. In one study, cognitive impairment was assessed using the MMSE [10], whereas in the remaining four studies, dementia was diagnosed using medical records. Diagnostic criteria for dementia included ICD-9 [30,32], ICD-10 [33], and Read codes [31]. Two studies defined dementia as Alzheimer’s disease [31,33], one study excluded Alzheimer’s disease [32], and one study included all types of dementia, including Alzheimer’s disease, vascular dementia, and non-vascular dementia [30]. All five studies adjusted for sex, age, and cardiovascular disease. In addition, three studies adjusted for diabetes or blood glucose levels [10,30,33]. Newcastle–Ottawa Scale scores ranged from 7 to 9 across the five studies, indicating a low risk of bias.

Evidence across the five prospective cohort studies was heterogeneous (*I*^2^ = 96.7%, Q = 152.3, *p* < 0.001). Two large-scale studies, each with a sample size greater than 140,000, identified a significant inverse association between baseline gout and subsequent dementia risk [30,31]. In a longitudinal study with a follow-up period exceeding 20 years, sex-specific outcomes were observed: female participants with gout had a significantly lower risk of MCI, whereas no significant association was observed among male participants [10]. Conversely, other studies reported conflicting findings. One study reported no significant effect on dementia incidence after 11 years of follow-up [33], whereas the largest cohort study found that a gout diagnosis was associated with a significantly increased risk of dementia over a six-year period [32].

## 4. Discussion

In this study, the systematic review did not identify a consistent association between hyperuricemia and the risk of dementia or cognitive decline. However, the cohort study showed that an increase in serum uric acid levels from normal status to hyperuricemia was associated with a lower risk of MCI, as reported in Table 2. This association remained statistically significant after full covariate adjustment. Nevertheless, the hyperuricemia and normal uric acid groups differed substantially at baseline in metabolic, demographic, and cognitive characteristics. Although these factors were adjusted for in the Cox regression models, residual confounding cannot be excluded. Therefore, the observed association should not be interpreted as direct evidence of a protective effect of hyperuricemia.

The relatively high cumulative MCI incidence warrants contextualization. All 1013 cases represent truly incident MCI, as participants with baseline MMSE scores below 27 were explicitly excluded at baseline. The Taiwan Biobank cohort was older, predominantly female, and urban-dwelling, all of which are established MCI risk factors in the elderly population [34,35], and likely contributed to the high incidence observed. Although a nationwide Taiwanese survey reported an MCI prevalence of 18.76% in adults aged ≥65 years [36], that study was conducted between December 2011 and March 2013, and had a lower participation rate, limiting direct comparability with the present findings. Furthermore, MCI prevalence has increased over time, likely reflecting rising rates of chronic diseases, including metabolic syndrome, diabetes, and cardiovascular disease, as well as increasing rates of depression and physical inactivity [37].

In the sensitivity analysis, results based on the US criteria for hyperuricemia were similar to those based on the Chinese criteria. The US definition of hyperuricemia is based on evidence that serum uric acid levels ≥ 6 mg/dL in women and ≥7 mg/dL in men are associated with significantly higher cardiovascular mortality [17]. Cognitive impairment is strongly associated with cardiovascular disease, and both conditions share several risk factors, including age and metabolic syndrome [38,39,40]. However, baseline serum uric acid levels cannot fully reflect longitudinal variation. Therefore, the cohort findings of the present study provide additional evidence that may help address gaps identified in the systematic review.

Limited studies have examined the relationship between hyperuricemia and cognitive decline. A 2013 meta-analysis found no significant association between serum uric acid levels and MCI risk [41]. However, this analysis included only two studies with a total of 129 participants. In contrast, several later studies reported significant associations between higher serum uric acid levels and lower MCI risk. In a cross-sectional study conducted in Beijing, China, involving 2102 community-dwelling older adults, higher serum uric acid levels were associated with a significantly lower risk of MCI [42]. In a systematic review, Yu and colleagues suggested that serum uric acid may have protective effects against MCI development [43]. Chen and colleagues followed 3103 community-dwelling older adults aged ≥65 years in China through the Healthy Aging and Biomarkers Cohort Study over a nine-year period and found that participants in the highest quartile of serum uric acid levels had a significantly lower risk of MCI than those in the lowest quartile [44]. However, these studies did not examine the effect of longitudinal changes in serum uric acid levels on MCI risk. To the best of our knowledge, the present study is the first to investigate the association between changes in serum uric acid levels and the risks of MCI and dementia in older adults.

The cohort study found that an increase in serum uric acid levels from normal status to hyperuricemia was associated with a lower risk of MCI. Wang and colleagues analyzed data from the China Health and Retirement Longitudinal Study and investigated changes in serum uric acid levels over seven years among participants aged ≥45 years and their effects on cognitive function [45]. They reported that an increase in serum uric acid levels from the initially normal range was associated with lower global cognitive scores (OR = 0.890, 95% CI = 0.834–0.950, *p* < 0.001). These results are inconsistent with the findings of the present study. Although both studies were conducted in Asian populations, the discrepancy may be attributable to differences in participant age and cognitive assessment tools. The present study focused on participants aged ≥60 years, whereas the study by Wang and colleagues included participants aged 45–60 years. MCI is a transitional stage between healthy aging and dementia [46]. Young-onset dementia occurs in individuals younger than 65 years, and its underlying mechanisms may differ from those of late-onset dementia [47]. In addition, Wang and colleagues used episodic memory and executive function tests to assess cognitive changes [45], whereas the present study used the MMSE, which is one of the most widely used tools for evaluating cognitive impairment [48,49].

The mechanisms by which serum uric acid may affect cognitive function remain unclear, but oxidative stress, inflammation, and amyloid-beta proteins have been proposed as potential pathways. Oxidative stress is one of the causes of cognitive decline, and metabolic syndrome may impair cognitive function through excessive reactive oxygen species (ROS) production [50,51]. Uric acid, the final oxidation product of purine metabolism in humans, is generated through xanthine oxidoreductase-mediated oxidation of hypoxanthine and xanthine [7]. Under oxidative stress, xanthine oxidoreductase shifts to its xanthine oxidase form, producing both uric acid and ROS, including superoxide anion and hydrogen peroxide. Paradoxically, uric acid itself is a major extracellular antioxidant, contributing approximately 50–60% of total plasma antioxidant capacity [52]. Uric acid scavenges hydroxyl radicals, peroxynitrite, and singlet oxygen and chelates transition metal ions such as iron and copper, thereby inhibiting metal-catalyzed oxidative reactions and hydroxyl radical generation [53,54,55]. Compared with other antioxidants, including ascorbic acid, alpha-tocopherol, and glutathione, uric acid primarily functions in the extracellular aqueous environment, particularly in plasma and cerebrospinal fluid [56]. However, the antioxidant effects of uric acid are concentration- and context-dependent. Excessively elevated intracellular uric acid levels may promote oxidative stress through xanthine oxidase activation, NADPH oxidase stimulation, and mitochondrial dysfunction [57]. Therefore, uric acid may exert both protective and detrimental effects depending on its concentration and physiological environment.

Although chronic hyperuricemia is the primary cause of gout and the two conditions are often discussed together [58,59], the development of gout varies substantially among individuals even at the same serum uric acid levels. Furthermore, gout is inflammatory in nature, whereas uric acid has dual antioxidant and pro-oxidant roles. These complexities likely contribute to the inconsistent findings between the cohort study and the systematic review.

In addition, higher serum uric acid levels have been associated with lower β-amyloid 1–42 levels and improved MMSE scores over a 2.9-year follow-up period [60]. These mechanisms may partly explain why increases in serum uric acid levels from normal levels to hyperuricemia were associated with a reduced risk of MCI in the present study. Nevertheless, serum uric acid levels are positively correlated with inflammatory markers, such as *C*-reactive protein and interleukin-6, which may contribute to endothelial dysfunction [61,62,63,64] and thereby increase the risk of vascular dementia [65]. This may explain why the present study did not identify a significant association between hyperuricemia and dementia risk.

Although participants with follow-up periods shorter than three years were excluded, the follow-up duration may be insufficient to evaluate dementia risk. Because the average duration of mild dementia has been estimated to be 5.6 years and dementia generally develops over a longer period than cognitive impairment [66], the limited follow-up may partly explain the inconsistent findings between MCI and dementia outcomes. Thus, the present results may reflect only the short-term association between hyperuricemia transitions and cognitive impairment in older adults without dementia. Longer-term studies are needed to clarify the effects of hyperuricemia transitions on dementia risk.

The five included studies showed considerable heterogeneity. Because formal subgroup analyses were not feasible, heterogeneity was explored narratively. Differences in gout definitions, including ICD-9 [30,32], ICD-10 [33], Read codes [31], and self-reported gout [10], as well as differences in cognitive outcomes, participant age, follow-up duration, and covariate adjustment, may explain the inconsistent findings.

The systematic review used the Newcastle–Ottawa Scale to assess risk of bias and found that all five included studies were of high quality. However, several factors may still contribute to potential bias. For example, one study relied on self-reported outcomes, which may have been influenced by participants’ memory [10]. In addition, gout was defined using ICD-9 [30,32], ICD-10 [33], or Read codes [31] in the other included studies. Although ICD-9 and ICD-10 are widely recognized and globally adopted disease classification systems, medical record-based ICD-9 diagnoses of gout may have limited validity [67], as observed in studies of male veterans [68]. Future meta-analyses should include more cohort studies with ICD-10-based gout diagnoses to reduce potential bias.

The exploratory mediation analysis showed no statistically significant indirect effect for any of the three candidate mediators, as reported in the Section 3. These findings suggest that the observed association was not significantly mediated through these metabolic factors in the present analysis. However, because mediator selection was partly data-driven, the mediation analysis may have been underpowered, and other biologically relevant mediators, such as oxidative stress markers and neuroinflammatory pathways, were unavailable, these null findings should be interpreted with caution.

The restricted cubic spline analysis confirmed a significant linear dose–response association between serum uric acid levels and MCI risk, consistent with the Cox models shown in Table 2 and Table 3. Nevertheless, the wide confidence intervals at the extremes of the distribution and the attenuation of the persistent hyperuricemia effect to non-significance after full adjustment (Table 2) suggest the possible influence of unmeasured confounders, as discussed in the limitations below.

This study has several limitations. First, a major limitation was the lack of data on urate-lowering medications, diuretic use, renal function, and socioeconomic indicators beyond education level, including income, occupation, and wealth, all of which may strongly influence both serum uric acid levels and cognitive outcomes. For example, lower socioeconomic status may independently associate with reduced healthcare access and greater cardiovascular burden [69], potentially confounding the observed association. Furthermore, socioeconomic differences across gout populations in different countries may have contributed to the heterogeneous findings observed in the systematic review [70]. Therefore, residual confounding cannot be excluded, and the observed association between hyperuricemia and lower MCI risk should be interpreted with caution. Second, the mean follow-up duration may have been insufficient to evaluate long-term dementia risk, and the relatively small number of participants who met the dementia threshold during follow-up may have underpowered the analysis in terms of detecting significant associations with dementia. Third, data on brain imaging, oxidative stress markers, and dietary patterns were unavailable. Therefore, we could not fully explore potential neuroanatomical, oxidative, or lifestyle-related mechanisms underlying the association between serum uric acid changes and cognitive outcomes. Fourth, the systematic review included only five prospective cohort studies. Although all included studies were considered high quality, substantial heterogeneity was observed, and the limited number of studies prevented formal subgroup analyses by outcome type, sex, geographic region, follow-up duration, or hyperuricemia definition. Fifth, the mediation analyses may have been underpowered, and residual confounding from unmeasured variables remains possible. Sixth, generalizability is limited by the demographic profile of the Taiwan Biobank cohort, which was restricted to Taiwanese community-dwelling older adults within a universal healthcare system and was older, predominantly female, and urban-dwelling compared with the general Taiwanese population. These characteristics are established MCI risk factors in this population [34,35] and likely contributed to the relatively high cumulative MCI incidence observed, limiting applicability to populations with different demographic profiles. Additionally, differences in gout diagnostic practices across healthcare systems and ethnicities, and known ethnic differences in uric acid metabolism and renal urate handling, further restrict generalizability. Future studies should include longer follow-up periods, medication profiles, renal function indices, dietary assessments, oxidative stress markers, and neuroimaging data to clarify the causal relationship between hyperuricemia and cognitive outcomes.

Clinically, although higher or increasing serum uric acid levels were associated with a lower risk of MCI in the cohort study, these findings should not be interpreted as evidence to intentionally increase serum uric acid levels or withhold urate-lowering therapy. Hyperuricemia remains an established risk factor for gout, cardiovascular disease, chronic kidney disease, and metabolic disorders. Therefore, urate-lowering treatment should continue to follow current clinical guidelines and individualized patient assessment.

## 5. Conclusions

In conclusion, an increase in serum uric acid levels from normal status to hyperuricemia was associated with a lower risk of MCI, but not dementia, in older adults. However, the accompanying systematic review of five prospective cohort studies yielded inconsistent findings, suggesting that the relationship between hyperuricemia and cognitive outcomes remains uncertain. These findings contribute to the current evidence on serum uric acid and cognitive impairment, but they should be interpreted cautiously because of potential residual confounding and limited follow-up duration. Importantly, the observed association should not be translated into clinical strategies aimed at increasing serum uric acid levels. Further well-designed prospective studies with longer follow-up, comprehensive medication data, renal function assessment, dietary information, and neuroimaging are needed to clarify the causal relationship between hyperuricemia and the risks of MCI and dementia [10].

## Figures and Tables

**Table 1:** Baseline characteristics of participants by hyperuricemia.

	Normal Uric Acid (*n* = 1519)	Hyperuricemia (*n* = 440)	*p* Value
Age (years)	64.38 ± 2.81	64.59 ± 2.83	0.178
MMSE *	**24.74 ± 1.56**	**24.53 ± 1.69**	**0.019**
Body mass index (kg/m^2^) *	**24.09 ± 3.10**	**26.32 ± 3.65**	**<0.001**
Waist–hip ratio *	**0.90 ± 0.07**	**0.91 ± 0.06**	**<0.001**
Systolic blood pressure (mmHg) *	**126.60 ± 17.64**	**130.81 ± 18.43**	**<0.001**
Diastolic blood pressure (mmHg) *	**72.95 ± 9.76**	**75.01 ± 10.10**	**<0.001**
Serum uric acid (mg/dL) *	**5.17 ± 0.96**	**7.32 ± 0.96**	**<0.001**
Serum HDL-C (mg/dL) *	**55.23 ± 13.79**	**49.44 ± 11.15**	**<0.001**
Serum triglyceride (mg/dL) *	**109.37 ± 60.21**	**145.18 ± 88.20**	**<0.001**
Fasting glucose (mg/dL)	**101.11 ± 23.95**	**103.17 ± 24.10**	**0.114**
HbA1c (%) *	**5.98 ± 0.89**	**6.11 ± 0.92**	**0.006**
Sex *			**<0.001**
**Male**	**36.3%**	**30.0%**	
**Female**	**63.7%**	**70.0%**	
Level of education *			**0.001**
**Primary school (≤6 years)**	**23.5%**	**16.8%**	
**Secondary school (7–12 years)**	**39.5%**	**38.0%**	
**University and above (>12 years)**	**37.0%**	**45.2%**	
Coronary artery disease			0.390
Yes	2.4%	3.2%	
No	97.6%	96.8%	
Diabetes *			**0.012**
**Yes**	**17.4%**	**22.7%**	
**No**	**82.6%**	**77.3%**	
Depression			1.000
Yes	3.2%	3.2%	
No	96.8%	96.8%	
Cigarette smoking *			**<0.001**
**Yes**	**23.8%**	**20.5%**	
**No**	**76.2%**	**79.5%**	
Living alone			1.000
Yes	8.7%	8.6%	
No	91.3%	91.4%	
Physical activity			0.310
Yes	64.6%	61.8%	
No	35.4%	38.2%	
Menopause *			**<0.001**
**Yes**	**25.6%**	**50.9%**	
**No**	**74.4%**	**49.1%**	

HDL-C: high density lipoprotein cholesterol. * *p* < 0.05 indicates a significant difference between the hyperuricemia and normal uric acid groups. Bold values indicate statistically significant differences (*p* < 0.05).

**Table 2:** Comparison of hazard ratios for mild cognitive impairment in different transitioning groups at baseline and follow-up.

Variables	*n* (%)	MCI Cases *n* (%)	Model 1		Model 2		Model 3	
			HR (95% CI)	*p* Value	HR (95% CI)	*p* Value	HR (95% CI)	*p* Value
Normal → Normal	1340 (68.4)	738 (55.1)	Ref.	Ref.	Ref.	Ref.	Ref.	Ref.
Normal → Hyperuricemia	179 (9.1)	82 (45.8)	0.72 (0.57–0.90)	0.005	0.75 (0.59–0.94)	0.012	0.75 (0.59–0.94)	0.013
Hyperuricemia → Normal	194 (9.9)	81 (41.8)	0.80 (0.64–1.01)	0.064	0.85 (0.67–1.07)	0.156	0.85 (0.68–1.08)	0.188
Hyperuricemia → Hyperuricemia	246 (12.6)	112 (45.5)	0.78 (0.64–0.95)	0.014	0.87 (0.71–1.06)	0.177	0.89 (0.73–1.10)	0.289

Model 1: Unadjusted. Model 2: Adjusted for age, sex, and education level. Model 3: Model 2 plus smoking status, living alone, overweight or obesity, and physical activity, and a history of coronary artery disease, diabetes, and depression.

**Table 3:** Comparison of hazard ratios for mild cognitive impairment in different transitioning groups at baseline and follow-up (US criteria).

Variables	*n* (%)	MCI Cases *n* (%)	Model 1		Model 2		Model 3	
			HR (95% CI)	*p* Value	HR (95% CI)	*p* Value	HR (95% CI)	*p* Value
Normal → Normal	1298 (66.3)	711 (54.8)	Ref.	Ref.	Ref.	Ref.	Ref.	Ref.
Normal → Hyperuricemia	191 (9.7)	89 (46.6)	0.76 (0.61–0.94)	0.014	0.77 (0.62–0.96)	0.022	0.77 (0.62–0.96)	0.022
Hyperuricemia → Normal	199 (10.2)	88 (44.2)	0.81 (0.65–1.01)	0.063	0.83 (0.67–1.04)	0.104	0.84 (0.67–1.05)	0.129
Hyperuricemia → Hyperuricemia	271 (13.8)	125 (46.1)	0.78 (0.64–0.94)	0.010	0.87 (0.72–1.05)	0.139	0.88 (0.72–1.07)	0.206

Model 1: Unadjusted. Model 2: Adjusted for age, sex, and education level. Model 3: Model 2 plus smoking status, living alone, overweight or obesity, and physical activity, and a history of coronary artery disease, diabetes, and depression.

**Table 4:** Characteristics of studies included in systematic review.

Author, Year	Country	Age at Baseline, Years	Men, %	Number of Participants	Hyperuricemia Definition	Outcomes Assessed	Covariates Adjusted for	Follow-Up, Years	Result	Score
Hong, 2015 [30]	Taiwan	≥50	63.3	143,511	ICD-9 (gout)	Dementia	Age, sex, diabetes, hypertension, hyperlipidemia, heart failure, coronary artery disease, chronic obstructive pulmonary disease, asthma, stroke, malignancy, chronic kidney disease, arrhythmia, Parkinson’s disease	4.3 ± 2.1 (gout cases), 4.4 ± 2.0 (non-gout controls)	↓ risk	8/9
Lu, 2016 [31]	UK	≥40	71.0	298,029	READ code (gout)	Dementia	Age, gender, entry time, BMI, smoking, alcohol use, physician visits, social deprivation index, comorbidities and medication use	5 (median)	↓ risk	7/9
Singh and Cleveland, 2018 [32]	USA	≥65	42.6	1,712,821	ICD-9 (gout)	Dementia	Age, sex, race, each of the 17 Charlson-Romano comorbidities, hypertension, hyperlipidemia, coronary artery disease, medications for cardiovascular diseases and for urate-lowering therapies for gout	6	↑ risk	8/9
Lee, 2023 [33]	Korea	≥40	80.1	90,395	ICD-10 (gout)	Dementia	Age, sex, income, residential area, obesity, smoking status, alcohol consumption, SBP, DBP, fasting blood glucose, total cholesterol, Charlson comorbidity index scores, Parkinson’s disease	11	Non-significant	9/9
Tan, 2023 [10]	Singapore	≥45	40.8	16,948	Self-reported gout	Cognitive impairment	Age at MMSE measurement, sex, dialect group, education level, smoking status, drinking status, physical activity, BMI, AHEI-2010 score, history of hypertension, diabetes, coronary artery disease and stroke	The longest duration was >20	↓ risk in female participants, but it was non-significant in male participants	7/9

AHEI-2010: Alternative Healthy Eating Index 2010, BMI: body mass index, DBP: diastolic blood pressure, MMSE: Mini-Mental State Examination, SBP: systolic blood pressure. ↑ indicates increased risk; ↓ indicates decreased risk.

## Data Availability

Cohort study: Restrictions apply to the availability of these data. Data were obtained from a third-party database (Taiwan Biobank) and are available from the data custodian with the permission of the third party. Due to data protection and privacy regulations, the authors are not per-mitted to share the raw data.

## Supplementary Materials

The following supporting information can be downloaded at: https://www.mdpi.com/article/10.3390/nu18111813/s1, Table S1. Full search strategy for each database.

## Abbreviations

The following abbreviations are used in this manuscript:

CIs	Confidence intervals
HRs	Hazard ratios
ICD	International Classification of Diseases
MCI	Mild cognitive impairment
MMSE	Mini-Mental State Examination
