# Supplementary material for: The Effect of Hyperuricemia on Cognitive Impairment: A Cohort Study and Systematic Review
Source: Nutrients. 2026 Jun 4;18(11):1813. doi: 10.3390/nu18111813 (PMC13258673; doi:10.3390/nu18111813)
Supplement: Supplementary file 1 [file nutrients-18-01813-s001.zip › nutrients-4314477-supplementary.pdf]

Table S1. Full search strategy for each database

Database	Platform	Search string	Filters	Date searched	Records retrieved
MEDLINE	Ovid	Step 1. exp Hyperuricemia/ OR hyperuricemia.mp. OR hyperuricaemia.mp. OR uric acid.mp. OR exp Gout/ OR gout.mp. OR urate.mp.<br>Step 2. exp Dementia/ OR dementia.mp. OR exp Cognitive Dysfunction/ OR cognitive impairment.mp. OR mild cognitive impairment.mp. OR MCI.mp. OR exp Alzheimer Disease/ OR Alzheimer*.mp. OR cognitive decline.mp. OR cognitive function.mp.<br>Step 3. exp Cohort Studies/ OR cohort study.mp. OR longitudinal study.mp. OR exp Longitudinal Studies/ OR prospective study.mp. OR exp Prospective Studies/ OR follow-up study.mp.<br>Step 4. 1 AND 2 AND 3	English	May 1, 2026	N = 110
PubMed	NLM	("Hyperuricemia"[MeSH] OR "hyperuricemia"[tiab] OR "hyperuricaemia"[tiab] OR "uric	English	May 1, 2026	N = 65

		acid"[tiab] OR "gout"[MeSH] OR "gout"[tiab] OR "urate"[tiab])<br>AND<br>("Dementia"[MeSH] OR "dementia"[tiab] OR "Cognitive Dysfunction"[MeSH] OR "cognitive impairment"[tiab] OR "mild cognitive impairment"[tiab] OR "MCI"[tiab] OR "Alzheimer's disease"[tiab] OR "cognitive decline"[tiab] OR "cognitive function"[tiab])<br>AND<br>("cohort study"[tiab] OR "longitudinal study"[tiab] OR "prospective study"[tiab] OR "follow-up study"[tiab])			
Embase	Elsevier	Line 1: 'hyperuricemia'/exp OR 'hyperuricemia':ti,ab OR 'hyperuricaemia':ti,ab OR 'uric acid'/exp OR 'uric acid':ti,ab OR 'gout'/exp OR 'gout':ti,ab OR 'urate':ti,ab<br>Line 2: 'dementia'/exp OR 'dementia':ti,ab OR 'cognitive impairment'/exp OR 'cognitive impairment':ti,ab OR 'mild	English	May 1, 2026	N = 605

		cognitive impairment':ti,ab OR 'MCI':ti,ab OR 'Alzheimer disease'/exp OR 'Alzheimer disease':ti,ab OR 'cognitive decline':ti,ab OR 'cognitive dysfunction'/exp OR 'cognitive dysfunction':ti,ab OR 'cognitive function':ti,ab<br>Line 3: 'cohort analysis'/exp OR 'cohort study':ti,ab OR 'longitudinal study'/exp OR 'longitudinal study':ti,ab OR 'prospective study':ti,ab OR 'follow up study'/exp OR 'follow-up':ti,ab<br>Final: Line 1 AND Line 2 AND Line 3			
Web of Science	Clarivate	Step 1. TS=("hyperuricemia" OR "hyperuricaemia" OR "uric acid" OR "serum uric acid" OR "gout" OR "urate" OR "uricemia")<br>Step 2. TS=("dementia" OR "cognitive impairment" OR "mild cognitive impairment" OR "MCI" OR "Alzheimer" OR "cognitive decline" OR "cognitive dysfunction" OR "cognitive function" OR	None	May 1, 2026	N = 114

		"cognition" OR "neurocognitive")<br>Step 3. TS=("cohort study" OR "cohort studies" OR "longitudinal study" OR "longitudinal studies" OR "prospective study" OR "prospective studies" OR "prospective cohort" OR "follow-up study")<br>Step 4. #1 AND #2 AND #3			
Scopus	Elsevier	TITLE-ABS-KEY("hyperuricemia" OR "hyperuricaemia" OR "uric acid" OR "serum uric acid" OR "gout" OR "urate") AND TITLE-ABS-KEY("dementia" OR "cognitive impairment" OR "mild cognitive impairment" OR "MCI" OR "Alzheimer*" OR "cognitive decline" OR "cognitive dysfunction" OR "cognitive function") AND ALL("cohort study" OR "longitudinal study" OR "prospective study" OR "prospective cohort" OR "follow-up study")	English	May 1, 2026	N = 999
Cochrane Library (CENTRAL)	cochranelibrary.com	Step 1. hyperuricemi* OR hyperuricaemi* OR (uric NEXT acid*) OR (serum NEXT	None	May 1, 2026	N = 116

		uric NEXT acid*) OR gout OR urate*<br>Step 2. dement* OR (cognitive NEXT impairment*) OR (mild NEXT cognitive NEXT impairment*) OR MCI OR Alzheimer* OR (cognitive NEXT declin*) OR (cognitive NEXT dysfunction*) OR (cognitive NEXT function*)<br>Step 3. #1 AND #2			
Airiti Library	airitilibrary.com	(高尿酸血症 OR 尿酸 OR 血清尿酸 OR 痛風 OR 高尿酸) AND (失智症 OR 認知障礙 OR 輕度認知障礙 OR 輕度認知功能障礙 OR 阿茲海默症 OR 認知功能下降 OR 認知功能 OR 認知衰退) AND (世代研究 OR 隊列研究 OR 群組研究 OR 梯次研究 OR 縱貫性研究 OR 前瞻性研究 OR 追蹤研究 OR 前瞻性世代研究)	Chinese or English	May 1, 2026	N = 1
National Digital Library of Theses and Dissertations in Taiwan	National Central Library (ndltd.ncl.edu.tw)	Step 1. (高尿酸血症 OR 尿酸 OR 血清尿酸 OR 痛風 OR 高尿酸)<br>Step 2. (失智症 OR 認知障礙 OR 輕度認知障礙 OR 輕度認知功能障礙 OR 阿茲海默症 OR 認知功能下降 OR 認知功能 OR 認知衰退)	Chinese	May 1, 2026	N = 4

		Step 3. (世代研究 OR 隊列研究 OR 群組研究 OR 梯次研究 OR 縱貫性研究 OR 前瞻性研究 OR 追蹤研究 OR 前瞻性世代研究)<br>Step 4. #1 and #2 and #3			
